# Report of Seven Cases of Puerperal Peritonitis

**Published:** 1856-12

**Authors:** Robt. K. Smith

**Affiliations:** Late Chief Resident Physician, Philadelphia Hospital


*Report of seven cases of Puerperal Peritonitis.* By ROBT. K. SMITH, M. D., Late Chief Resident Physician, Philadelphia Hospital.

The following seven cases of puerperal peritonitis are a continuation of the series reported in the April No. of the Examiner. Except the first, they all occurred in the obstetrical wards of the Hospital connected with the Philadelphia Alms House, and constituted a part of the number included in the prevailing epidemic.

The two first in the present report were under treatment at the time I closed my communication upon that subject, (referred to in the April number,) and resulted as I then supposed. The remaining five were subsequent cases, and with them terminated the disease in the Institution.

The first case in the present report was a lady, residing five miles from the Hospital; in a healthy locality; where there had been no case of puerperal fever, and to whom I had been called, in consultation, after delivery, in consequence of an adherent placenta. The report exhibits my professional connexion therewith, and it is for the profession to decide, whether the probabilities favor, in this instance, the communicability of the disease. I will remark, however, that in the Hospital, the eight attending physicians attended the obstetrical patients in rotation, in the alphabetical order of their names; and unless there was some abnormal condition, I was rarely called upon for assistance. During the prevalence of this disease, this assistance was rendered upon several occasions, and from its commencement until its termination, seventy women in all were delivered; many of whom escaped an attack, although subjected to such manual assistance, and occupying the same rooms, often adjoining beds, to the fever patients. Commencing with the first and including the last case of puerperal fever, there were seventy obstetrical cases in the Hospital, and out of the seventy, thirty-two had the fever. In two of these cases the operation of craniotomy was performed; one had the fever and died; and the other escaped an attack. Every instrumental case was treated during labor by the chief medical officer, when the institution was under my control; I am thus particular in a description of its management for the purpose of showing that those who had the disease, and those who escaped, were attended indiscriminately by the medical staff of the house.

From reliable information received, a lady relative of the first case in this report, who attended the funeral, and who was shortly after delivered, died in childbed of the same disease. This lady lived several miles further in the country, and from what I can learn, it was the only case of the kind in that vicinity.

My own experience does not justify the belief that there is a specific contagion in puerperal fever, and yet I am free to admit, that if this case had occurred without my interference, I should have felt much more comfortable.*

* As regards the communicability of the disease, we are allowed to state the following curious experiment related to us by a friend, an obstetrician of large practice, who does not, however, wish to have his name mentioned. He was in attendance upon four women, ill with puerperal fever, three of whose residences were in the same block of houses, within a few doors of each other and on the same side of the street. He had just left the house of one of them, a poor woman, who was seriously ill, where he had been passing a considerable portion of time in the performance of various kind offices, such as putting clean clothes upon her, dressing her blister and drawing off her water, when he was called to see a patient in labor on the opposite side of the way. He at first refused to go, informing the husband that he had several cases of child-bed fever under his care, had just left one, and that the disease was believed to be contagious by many physicians. The husband, however, admitting of no denial, he crossed over the street and took charge of the case. He found the woman in active labor, and

*Case* 1*st*. Mrs. ———, a married lady; born in Pa.; aet. 36; healthy, and of robust constitution; sanguine bilious temperament; sixth pregnancy; was delivered March 11th of twin boys, after an easy labor. I was called in consultation in the afternoon, and removed the placenta by introducing my hand within the uterus. This organ contracted readily; there was no hæmorrhage; the patient was lively, and I left her comfortable.

In consequence of the prevalence of puerperal fever in the Hospital I suggested to the attending physician the necessity of the most careful watchfulness; and advised the most prompt and energetic treatment in the event of abdominal pain or an accelerated pulse. The following day, (the 12th,) her physician saw her five or six times, and reported no unfavorable symptoms. On the evening of the 13th for the first time, he remarked an increased frequency of the pulse, and said she complained of slight uterine soreness and pain, when questioned upon this point. He had seen her frequently through the day, and these were the first noticeable symptoms. An opiate was prescribed, and she passed a comfortable night.

14th. The doctor rode over to the Hospital and reported her condition; was desirous that I should see her; said there was nothing alarming in her appearance; that she had but little pain; but her pulse was 132, and she looked pale. My engagements prevented a visit, but I urged him to take with him a leecher, apply 150 to her abdomen, to be followed by a succession of warm poultices, and to give her a grain of calomel and two grains of opium every hour until the pain was relieved, and a favorable change took place.

after anointing his index finger with lard, made an examination. Finding that the labor would not be over for some time, and being somewhat of a disbeliever in the contagious nature of the disease, he resolved to try an *experimentum crucis*, the result of which should determine, to his own mind at least, the question of its communicability. This was to place his whole hand, which we should mention however had been washed before leaving the house on the opposite side, in the vagina of his patient, and leave it there for some time. He accordingly kept it there for an hour and a half. No ill effect followed this venturesome essay, as the woman made in all respects a quick recovery. He also informed us that during his attendance upon these cases 18 women were delivered by him, all of whom did well.—*Ed.*

15th. Saw the patient at 8 A. M. She told me the leeching had relieved the pain almost immediately, and she had suffered but little since. She had been wakeful through the night; looked pale and anxious; skin cool and moist; pulse small, 130; lochia almost suppressed; abdomen tympanitic, and very great tenderness upon pressure throughout its whole extent; dorsal decubitus and legs extended. The treatment of yesterday continued, with carb. ammonia and brandy; and an enema of oil and turpentine directed. Saw the patient again at night; had been vomiting occasionally, was more feeble; tympanitis increasing; pulse 136, and pain aggravated. A turpentine enema was administered, and twice repeated through the night: morphia was substituted for the calomel and opium. A carminative was given, and in the morning the vomiting had ceased; her pulse was less frequent, and the tympanitis considerably lessened. She had slept none, but I thought there was a slight general improvement, and left her to return again during the afternoon, (the 16th.)

Saw her again in the afternoon; no change; her physician said she had continued much the same all day; vomiting had not recurred; and the treatment was continued. A grain of morphia had been given every hour, and although it had not procured sleep, the pain was absent when the patient was undisturbed.

17th. Patient sinking; stimulants given freely all day; saw her in the evening dying; died before midnight; no post mortem.

The twin boys of this patient both died in convulsions, one a day or two before the mother, and the other before she was buried.

In this case it will be observed there was no general depletion; and the rigid antiphlogistic treatment resorted to in Mrs. H.'s case (No. X. published in the April number) was not adopted. The patient was a woman more plethoric and robust; accustomed to more active exercise, and altogether one whose system in health would have borne more general depletion. Her apparent prostrate condition, and the lapse of time from the appearance of the first symptoms, until I saw her, together with the fact that I was not alone in the case, made me hesitate to adopt it.

This lady had been my former patient; I had attended her in three confinements, one of which was a twelve months' pregnancy, resulting in the birth of a mole, an organized muscular-fibrous mass, weighing about a pound, and unaccompanied by the secundines. This labor was attended by all the usual phenomena, until the body was expelled; and her after recovery did not differ from ordinary cases. This event in the history of the patient is now only referred to, as an anomalous fact, that there was no expulsive effort of nature at the usual termination of utero-gestation (9 months) and no symptom or accident that would satisfactorily account for this extended period of pregnancy.

*Case* 2*d.* Mary Ann Gibson (deaf mute); born in Ireland; æt. 25; married; rather asthenic; delivered of her first child February 18th; was getting over her confinement remarkably well until the night of March 10th, nearly three weeks after delivery, when she had a chill and complained of headache. Saw her at 10 A. M. the 11th; had headache; pulse 146 and weak; sharp abdominal pains; great tenderness on pressure in the iliac regions; and slight tympanitis. Ordered 250 leeches on abdomen, followed by hot fomentations of turpentine, gave her 10 grains of calomel and in five hours a dose of castor oil. Directed 15 grains of quinine and 5 ounces of brandy to be given during the day.

At 3 P. M. patient in much the same condition; had been leeched and fomentations applied. 8 P. M. Still has pain and tympanitis; ordered a blister to the abdomen to be dressed with morphia grs. ii. and a ½ grain of morph. to be given every hour until she slept.

12th. 9½ A. M. Had slept but little during the night; skin dry; fever; pulse 118; pain; slight tympanitis; evidently subsiding; continued the quinine: prescribed Liq. Potass. Cit. f.℥ix. Sp. Æth. Nit. f.ʒii. ℳ. A tablespoonful every two hours, and substituted 1 gr. opium every hour for the morphia, an injection of Ol. Ricini and a tablespoonful of turpentine was administered; she slept a little during the day.

8 P. M. Patient better; skin cool and moist; pulse 102 and more regular; bowels opened twice since morning; tongue slightly furred. Ordered a grain of calomel and a grain of opium every hour, when awake.

13th. Still improving; complains of but little pain; no fever; skin cool and pleasant; pulse about 83 and regular; continued treatment.

14th. 9½ A. M. No fever; pulse 80; no pain; slept well during the night; ordered the powders every two hours.

15th. Still better; no fever; discontinued treatment, and she continued to convalesce without a bad symptom. Child living.

This case is remarkable for the lapse of time intervening between delivery and the period of attack, as well as for the large amount of blood drawn locally by the leeches. Estimating six leeches to the ounce, there were nearly forty-two ounces of local blood-letting. The effect of this depletion was to reduce the pulse in 18 hours from 146 to 118, without exhausting the vital forces; and the rapid and steady improvement of the patient is the most convincing evidence of the propriety of the treatment.

*Case 3d.* Hannah Foley, born in Ireland; single; æt. 22; sthenic; first pregnancy; delivered March 30th of a dead boy after a labor of eleven hours.

April 1st. Patient had a chill last night, and complains of pain in the lower belly; cannot bear the slightest pressure; pulse 136; dry skin and red glazed tongue. Bled her at 8 this morning, ℥xxxiii. and ordered 150 leeches to the abdomen; gave calomel ten grains followed by a dose of castor oil, and the operation of the medicine to be assisted by an oil and turpentine injection. 6 P. M. Pain violent; gave opium 4 grains every hour, to be continued until she sleeps. Tympanitis appeared, and ordered the turpentine injection to be repeated.

2d. 7 A. M. Pulse 125; skin cool and moist; patient passed a restless night; pain almost gone; prescribed calomel ½ grain, opium 2 grains every hour.

The patient seems cheerful and better, and considering the amount of blood drawn by the lancet and leeches, has borne it remarkably well. 6 P. M. Hot dry skin, pulse 140; tympanitis excessive; restless and partially delirious; sleeps none; increased the opium to four grains every hour, and continued the injections three or four times during the night.

3d. Patient very ill; pulse barely distinguishable, and so rapid as to be scarcely able to note its frequency; gave her brandy and water until she died, which was in the afternoon. No post-mortem.

*Case 4th.* Ellen Dona; born in Ireland; æt. 23; third pregnancy; was delivered March 22nd. This woman had been in these wards of the institution upon a former occasion, and had also been an inmate of the lunatic asylum. She was insane when last admitted; frail constitution and anæmic. Seemed to be doing well until the morning of the 2nd of April, when she had a chill and violent abdominal pains. Bled her ℥xxx. and applied 200 leeches to the abdomen, followed by hot turpentine fomentations; pulse before bleeding 140. 6 P. M. Patient better, except the presence of tympanitis; ordered turpentine injection; and 3 grs. opium every hour until she sleeps.

3d. Pulse 110; pain much relieved; tympanitis greatly reduced; skin and tongue dry; prescribed nitrous powders and the opium.

4th. A great change for the better, pulse 90, skin and tongue moist; no pain. Continued treatment.

5th. Convalescent, suspended treatment, and she gradually recovered her strength, and was transferred to the Lunatic Asylum.

*Case 5th.* Mary Doland, born in Ireland; single; æt. 21. Delivered in the city, March 31st, and brought to the institution April 1st. A feeble anæmic woman; appears to have suffered from want and destitution.

April 4th. Had a slight chill in the morning at 7 o'clock; at M. tenderness in the iliac regions and pulse 124. Ordered 200 leeches, and hot poultices to the abdomen. Prescribed:—℞ Hyd. Chlor. Mit. grs. viii.; Potass. Nit. ʒiii; Tart. Antim. grs. ii; M. Div. chart. no. xvi; one every two hours, and Hyd. Chlor. Mit. grs. ii.; P. Opii. grs. ii. every alternate two hours until she sleeps. 7½ P. M. Leeches had been applied; tenderness entirely removed; abdomen slightly tympanitic; pulse 105; tongue moist and clean; bowels moved once. The patient continued without change, subjected to the same treatment, except that the calomel and opium powders were withdrawn, and the leeching not repeated, until the morning of the 7th, when the tenderness reappeared, and the tympanitis slightly increased; pulse 96; tongue brown but moist. Prescribed the calomel and opium again every two hours; gave an injection of oil of turpentine, and saw her again at

7 A. M. 8th. Patient rested well last night, tongue cleaner; pulse 88. Continued the powders.

9th. 3 A. M. Dr. Marshall was called up to see the patient, who was very restless; he gave her 60 drops of laudanum, and she became quiet, but slept none. No increased frequency of the pulse.

7 A. M. Patient quiet; no increased frequency of the pulse; no pain; continued treatment.

10th. 12 M. Pulse increased in frequency, 110; slight pain in the chest; face flushed. Had got out of bed during the night and exposed herself in the ward, when the assistant physician was called; prescribed an ounce of oil and neutral mixture every hour.

11th. Much better; discontinued medicine, and the patient gradually recovered her strength. This patient was in the institution in February when the fever was most fatal, and on account of the alarm felt by most of the pregnant women was one of those that had been discharged to seek other accommodations. After her recovery, she was, with her child, discharged from the House.

*Case 6th.* Margaret Gingling, born in Germany; æt. 26; single; a frail, anæmic woman in the last stage of phthisis pulmonalis; delivered, March 29th, of a boy; first pregnancy; was free from symptoms of fever until the morning of the 4th of April. Had a slight chill, and abdominal pain; pulse 128; ordered 200 leeches to abdomen, and hot poultices. Prescribed ℞. Calomel grs. viii.; P. Nit. Pot. ʒiii; Tart. Ant. grs. iii. Div. Chart. no. xxiv. One every two hours. 3 P. M. Leeches are being applied; abdomen slightly tympanitic; tenderness in epigastric region; anxious expression; pulse 110, weak and irregular.

5th. 10 A. M. Abdomen tympanitic; tenderness still present; ordered an oil and turpentine injection; pulse 102. Continued the powders.

6th. Tympanitis still present; continued the poultices; great tenderness under the ensiform cartilage, pulse 104; tongue furred and dry. 8 P. M. Pulse 100; no tympanitis; bowels moved freely.

7th. Pain and tympanitis gone; pulse 100, weak and irregular. Continued the powders.

8th. 7 A. M. Patient doing well; pulse 90; skin cool and moist; continued powders.

9th. 2 A. M. Dr. M. called to patient, who would not stay in bed; gave her a large dose of laudanum, (a teaspoonful); pulse small, and 85. She became quiet and went to sleep.

10th. All symptoms of peritonitis have disappeared; pulse 80: and patient very comfortable, except her cough; discontinued treatment. This patient gradually recovered her strength, and was transferred to the medical wards to be treated for phthisis; whence she was discharged from the Institution several weeks afterwards. Her child died on the 12th of April in convulsions.

*Case 7th.* Mary Ann McInrow, born in Ireland; æt. 25; married; second pregnancy; was delivered of a female child May 2nd; had a difficult labor; adhesion of the placenta and concealed hæmorrhage.

May 3rd. Had a slight chill last night; pulse at 7 A. M. 134; great abdominal soreness; pain; dry red tongue; tympanitic. Ordered 125 leeches to the belly; and an oil and turpentine injection. Prescribed 2 grs. calomel and 3 grs. opium every hour; after the leeching, pulse fell to 125; and the tenderness was much relieved.

4th. No pain; abdomen soft, and but slightly tympanitic; tongue clean and moist; pulse 120; great thirst; continued treatment.

5th. Patient much better; pulse 90; skin cool; no thirst. Continued powders.

6th. Patient convalescent; stopped treatment, and she gradually regained her strength. Child still in the House.

The whole number of deaths from puerperal fever during the epidemic was 18, out of 34 treated: 32 hospital patients, and two in private practice. Of the children born of those 34 women, 18 died of convulsions, and five were still-born.

In the report of the first 27 cases it was stated that erysipelas was prevalent in the surgical wards at the time the fever appeared in the obstetrical; and it is proper to say that these two diseases prevailed, subsided, and disappeared simultaneously.

The treatment was founded, not only upon what I believe to be correct principles, but upon former experience; limited it, is true, but nevertheless sufficiently satisfactory to secure my confidence. The results for hospital practice I confidently exhibit with every statistical table I have yet seen, and I believe they will compare favorably with any ever published. Adopting the views of Drs. Gordon, Armstrong, Hey and Tonnellé, and sustained by Dr. Meigs, that puerperal fever is an *inflammation*, often so violent and overwhelming as to almost paralyse the nervous system, producing symptoms so deceptive as to arrest the hand of even the boldest and most experienced practitioner; presenting appearances of nervous and vascular depression, which, as a *real primary* condition, would properly demand a system of treatment the very opposite to that upon which I have relied—I say, adopting these views and acting upon these principles, I have had the courage to bleed and starve into health those who seemed to have scarcely blood enough left to feed the flame that was consuming them.

In looking over the published Transactions of our State Medical Society, I see that some exception has been taken to this method of treatment, particularly as applied to my cases, by a gentleman in Schuylkill county; and we have from him the gratuitous suggestion that an opposite course might have secured different results. As this gentleman has not seen proper to state precisely this difference, I can say to him, that upon this point we fully agree, but it is equally my "*firm belief*" that this difference would have been much more disastrous to my patients, and much less creditable to myself.

If Dr. Brown had given the profession the benefit of his enlarged experience, by an accurate and honest detail of his cases, it would have been more satisfactory to them than to have the Transactions of the State Medical Society made the vehicle of a lame and dogmatical criticism; and more modest in him than to expect us to take a partial and imperfect description of a disease, which he chooses first to designate as puerperal fever, and afterwards speaks of as metritis—*making a distinction between them*—leaving his readers in doubt whether any *real* disease actually existed in the "pure invigorating atmosphere" of Schuylkill county, and to set this up as a parallel to the frightful epidemic prevailing in an alms-house hospital.

Such an exhibition of this gentleman's talents and intelligence is not very creditable, and is exceedingly egotistical, not to use a harsher expression.

The subjoined table exhibits the proportionate mortality in the various hospital epidemics of puerperal fever, of which I have any present account.

Churchill says the first undoubted epidemic of child-bed fever of which we have any account, occurred in the Hotel Dieu the winter of 1746. Out of twenty women confined in that year, scarcely one recovered.

In private practice, where the disease prevailed epidemically, we have the record of

| | | | |
|---|---|---|---|
| Dr. Gordon's Aberdeen cases, | 77, | 28 deaths, | 1791. |
| Mr. Hey's Yorkshire " | 14, | 11 " | 1810. |
| M. Botrel's Rennes " | 24, | 22 " | 1842–4. |

Dr. Wm. Hunter lost 31 out of 32 cases.

Dr. Robert Lee, London cases 160, 68 deaths.

| | | | | |
|---|---|---|---|---|
| In 1813, | Dumfries cases | 28, 27 deaths. | | |
| In 1823, | Leith " | 16, 16 | " | |
| | Inveresk " | 16, 16 | " | Dr. Kellie. |
| In 1841 to 1846, | Philad'a " | 95, 18 | " | Dr. Rutter. |

In 1853. in the village of Brakel, Minden, 13 cases, 12 deaths.

*Table of Hospital Cases.*

| Year. | Hospital. | No. of Cases. | No. of Deaths. | | Per cent. |
|---|---|---|---|---|---|
| 1764 | Dublin | 28 | 21 | or | 75 |
| 1770 | Westminster | 19 | 14 | | 73 |
| 1773 | Royal Infirm. Edinb'g, all died | | | | 100 |
| 1787 | Dublin | 11 | 7 | | 64 |
| 1788 | " | 17 | 14 | | 82 |
| 1814–15 | Edinburgh | 9 | 8 | | 88¾ |
| 1819–20 | Dublin | 192 | 86 | | 46 |
| 1828–29 | " | 88 | 56 | | 63⅔ |
| 1830 | Edinburgh | 18 | 16 | | 89 |
| 1832 | Penna. Hospital | 8 | 5 | | 62½ |
| 1833–34 | Vienna | 175 | 109 | | 62⅓ |
| 1835 | Dublin | 13 | 8 | | 61½ |
| 1845 | " | 14 | 10 | | 71 |
| 1845 | Penna. Hospital | 3 | 3 | | 100 |
| 1854–5 | Dublin | 38 | 21 | | 55⅓ |
| 1856 | Plilada., Blockley | 32 | 17 | | 53 |

